# The Genetic and Transcriptomic Nexus of Age-Related Hearing Loss and Alzheimer’s Disease

**DOI:** 10.3390/genes17070776

**Published:** 2026-06-30

**Authors:** Kaige Deng, Xu Deng, Siying Li, Shubin Fang, Fanqin Wei

**Affiliations:** 1 Department of Otorhinolaryngology, The First Affiliated Hospital, Sun Yat-sen University, Guangzhou 510062, China; dengkaige_cam@163.com; 2 Guangdong Medical University, Dongguan 523808, China; dengxu0106@163.com; 3 School of Medicine, South China University of Technology, Guangzhou 510006, China; lsybingbing@163.com

**Keywords:** age-related hearing loss, Alzheimer’s disease, causal inference, expression quantitative trait loci, genetic epidemiology, CSK

## Abstract

**Introduction**: Despite the recognized association between age-related hearing loss (ARHL) and Alzheimer’s disease (AD), the genetic relationship and shared transcriptional mechanisms between them remain largely unexplored. **Methods**: We systematically investigated the ARHL-AD axis using a multi-layered genomic strategy, incorporating causal inference, cis-eQTL mediation, Bayesian colocalization, and independent replication cohort validation. **Results**: Genetically predicted ARHL was associated with a reduced risk of overall AD and its early/late-onset subtypes, with no evidence of reverse causality in reverse MR analyses. Among 36 identified mediating genes, CSK showed the most consistent mediation signals across discovery and replication cohorts, although the replication was statistically partial (Sobel *p* = 0.078). Pathway analyses revealed that these genetic links predominantly involve Wnt signaling and endoplasmic reticulum protein processing. **Discussion**: Our integrative multi-omics findings suggest a potential genetic association between ARHL liability and AD risk. More importantly, we identified a prioritized CSK-driven transcriptional network, providing novel mechanistic insights and highlighting a candidate gene for future functional investigation in neurodegeneration.

## 1. Introduction

Age-related hearing loss (ARHL), also known as presbycusis, is one of the most common sensory impairments among older adults, while Alzheimer’s disease (AD) is the leading cause of dementia worldwide. With the rapid aging of the global population, both conditions have emerged as major public health challenges. Epidemiological evidence indicates that approximately 20% of the global population experiences some degree of hearing loss, and nearly 62% of affected individuals are older than 50 years [1]. Meanwhile, the global number of people living with dementia was estimated at 57 million in 2019 and is projected to increase to 153 million by 2050 [2], highlighting the growing societal and economic burden associated with neurodegenerative disorders.

Accumulating evidence suggests a close relationship between hearing loss and cognitive decline. Clinical and epidemiological studies, including large prospective cohort studies, have consistently reported that hearing impairment is associated with an elevated risk of dementia and Alzheimer’s disease [3,4,5]. Moreover, the risk of dementia appears to increase with the severity of hearing loss, suggesting a potential dose-response relationship between auditory impairment and cognitive decline [3]. These findings have led to the recognition of hearing loss as one of the most important potentially modifiable risk factors for dementia. However, although numerous observational studies have reported associations between hearing loss and Alzheimer’s disease, the biological mechanisms underlying this relationship remain largely unclear. In particular, it remains uncertain whether hearing loss directly contributes to AD pathogenesis or whether the observed associations are driven by shared aging-related processes or confounding factors. Furthermore, few studies have investigated the potential genetic mechanisms linking ARHL and AD, particularly at the level of gene expression regulation.

From a biological perspective, ARHL is considered a multifactorial disorder influenced by aging, environmental exposures, and genetic susceptibility. Multiple molecular mechanisms have been implicated in its pathogenesis, including oxidative stress, mitochondrial dysfunction, and chronic inflammation within the auditory system [6,7]. These processes may lead to progressive degeneration of cochlear hair cells, spiral ganglion neurons, and other components of the auditory pathway [8]. Despite these proposed mechanisms, the genetic architecture underlying ARHL and its potential connection with neurodegenerative diseases remain incompletely understood. Integrating genetic data with transcriptomic regulatory information may therefore provide new insights into the molecular pathways linking hearing loss and dementia.

Expression quantitative trait loci (eQTLs) represent genetic variants that influence gene expression levels and provide an important framework for linking genetic variation with downstream molecular phenotypes [9]. By integrating genetic variants with transcriptomic regulation, eQTL analyses can help identify genes whose expression is genetically regulated and potentially involved in disease development. Mendelian randomization (MR), which uses genetic variants as instrumental variables, has emerged as a powerful analytical approach for evaluating potential causal relationships between exposures and disease outcomes while minimizing confounding and reverse causation [10,11]. The integration of MR with eQTL data further enables investigation of disease mechanisms at the transcriptional regulatory level and facilitates the identification of candidate genes that may mediate associations between complex traits and diseases [12].

In the present study, we aimed to systematically investigate the genetic relationship between age-related hearing loss and Alzheimer’s disease using an integrative genomic framework. First, two-sample Mendelian randomization analyses were conducted to evaluate the potential causal effect of ARHL on AD and its clinical subtypes. Reverse Mendelian randomization analyses were subsequently performed to assess possible bidirectional relationships. We then incorporated cis-eQTL data to identify genetically regulated genes that may mediate the association between ARHL and AD. To strengthen causal inference, mediation analyses, Bayesian colocalization analyses, and independent replication using external eQTL datasets were performed. Finally, protein-protein interaction network construction and functional enrichment analyses were used to explore the biological functions and pathways associated with the identified mediating genes. Through this multi-layered analytical strategy, our study aims to provide novel genetic evidence and mechanistic insights into the relationship between ARHL and Alzheimer’s disease.

## 2. Methods

### 2.1. Study Design

This study was designed to systematically investigate the genetic relationship between age-related hearing loss (ARHL) and Alzheimer’s disease (AD) and to explore potential cis-regulatory mechanisms underlying this association. An integrative analytical framework combining Mendelian randomization (MR), mediation analysis, Bayesian colocalization, replication validation, protein-protein interaction (PPI) network construction, and functional enrichment analyses was implemented.

Initially, two-sample Mendelian randomization analyses were conducted to evaluate the causal effect of ARHL on overall AD as well as its clinical subtypes. Reverse Mendelian randomization analyses were further performed to assess potential bidirectional relationships and strengthen inference regarding causal directionality. Subsequently, cis-expression quantitative trait loci (cis-eQTLs) were used as genetic instruments to identify genetically regulated genes associated with ARHL and AD. Genes demonstrating significant associations with both traits were subjected to mediation analysis to evaluate whether their effects on AD were mediated through ARHL. To strengthen causal interpretation, Bayesian colocalization analyses were conducted to determine whether shared causal variants underlay the observed associations. Finally, prioritized genes were evaluated using replication analyses, PPI network construction, and Gene Ontology (GO) and Kyoto Encyclopedia of Genes and Genomes (KEGG) enrichment analyses to explore their biological relevance.

### 2.2. Data Sources

Summary-level genome-wide association study (GWAS) data for age-related hearing loss (ARHL) were obtained from a large-scale meta-analysis conducted in 2020 by the European Molecular Biology Laboratory-European Bioinformatics Institute (EMBL-EBI, https://www.ebi.ac.uk/). This dataset comprised 330,759 individuals of European ancestry and represents one of the largest publicly available genetic resources for ARHL (PMID: 32986727).

GWAS summary statistics for Alzheimer’s disease (AD) were obtained from the FinnGen R12 release (https://www.finngen.fi/en/access_results, accessed on 1 January 2026). Three AD phenotypes were included in the present study: overall Alzheimer’s disease (AD_WIDE), early-onset Alzheimer’s disease (AD_EO), and late-onset Alzheimer’s disease (AD_LO). All datasets were restricted to individuals of European ancestry to minimize potential bias due to population stratification.

The foundational details of these outcome datasets can be found in Table 1 and Supplementary Table S1.

### 2.3. Selection of Cis-eQTL Instruments

Cis-expression quantitative trait loci (cis-eQTLs) were obtained from the Genotype-Tissue Expression (GTEx) database (https://www.gtexportal.org/home/, accessed on 1 January 2026). The set of druggable genes was defined based on two complementary resources: (1) the updated “druggable genome” reported by Finan et al. [13], which provides a systematic compilation of genes encoding proteins amenable to modulation by small molecules or biologics; and (2) the Drug-Gene Interaction Database (DGIdb, https://www.dgidb.org/, accessed on 1 January 2026), a publicly accessible resource that aggregates druggability annotations from over 30 expert-curated sources. This combined gene set has been widely applied in systematic druggable genome-wide Mendelian randomization studies to prioritize targets with therapeutic translational potential [14,15]. To construct instrumental variables, we identified single nucleotide polymorphisms (SNPs) with a *p* < 5 × 10^−8^ located within ±100 kb of the transcription start site of each druggable gene, consistent with established strategies in large-scale Mendelian randomization studies. Standard quality control procedures were applied, including allele harmonization and removal of ambiguous variants. Linkage disequilibrium pruning was performed at an *r*^2^ threshold of <0.001 using European samples from the 1000 Genomes Project to ensure the independence of instrumental variants. After filtering, a total of 6903 cis-eQTLs were retained and harmonized with the GWAS summary statistics for age-related hearing loss (ARHL), which served as genetic instruments for subsequent Mendelian randomization and colocalization analyses [13,14,15] (Supplementary Table S2).

To validate the robustness of prioritized mediating genes, replication analyses were conducted using independent cis-eQTL data from the eQTLGen consortium (https://www.eqtlgen.org/cis-eqtls.html, accessed on 1 January 2026); additionally, tissue-specific cis-eQTL data from GTEx v8 brain regions (Brain-Hippocampus and Brain-Frontal Cortex) were also screened for the prioritized genes, following the same selection criteria (*p* < 5 × 10^−8^, ±100 kb of transcription start site, *r*^2^ < 0.001) to explore brain-specific regulatory signals. The same selection criteria and harmonization procedures were applied in the replication stage to ensure methodological consistency.

### 2.4. Mendelian Randomization Analysis

Two-sample Mendelian randomization analyses were performed to estimate causal effects using the “TwoSampleMR” package in R [16,17]. All statistical analyses were carried out using R 4.4.1 (the link to access it is https://www.r-project.org, accessed on 1 January 2026). A stringent significance threshold of *p* < 1 × 10^−5^ was used to determine significant causal associations in forward Mendelian randomization analyses of ARHL on AD phenotypes. This threshold was chosen to retain a sufficient number of instrumental variables for the ARHL exposure GWAS, as the number of genome-wide significant SNPs (*p* < 5 × 10^−8^) was limited. All selected instruments had F-statistics > 10, indicating adequate instrument strength to mitigate weak instrument bias. When only a single instrumental variant was available for a given exposure, the Wald ratio method was applied to estimate causal effects. When multiple independent variants were available, inverse variance weighted (IVW) analysis was used as the primary method.

Reverse two-sample Mendelian randomization analyses were additionally performed to assess the potential causal effect of AD on ARHL, so as to exclude reverse causality. Genetic instruments for three AD phenotypes (AD_WIDE, AD_EO, AD_LO) were constructed following the identical quality control criteria and analytical procedures applied in the forward MR analysis.

Heterogeneity among instrumental variables was assessed using Cochran’s Q statistic [11]. To evaluate potential horizontal pleiotropy, additional sensitivity analyses were conducted, including MR-Egger regression, weighted median, weighted mode, and simple mode approaches [18,19]. Leave-one-out sensitivity analyses were additionally performed to assess whether any single instrumental variant disproportionately influenced the causal estimates. The consistency of effect estimates across different MR methods was examined to assess robustness. IVW estimates were used as the primary measure of causal effect, while weighted median and MR-Egger were applied as complementary sensitivity analyses to assess robustness.

False discovery rate (FDR) correction was applied where appropriate to account for multiple testing.

### 2.5. Mediation Analysis

For mediation analysis, cis-eQTLs were first evaluated for their associations with ARHL and AD phenotypes using Mendelian randomization. eQTLs significantly associated with ARHL and at least one AD phenotype after FDR correction were considered candidate mediators.

The indirect effect of each candidate eQTL on AD through ARHL was calculated as the product of the eQTL-ARHL and ARHL-AD effect estimates. Sobel tests were performed to assess the statistical significance of the mediation effect. The proportion mediated was calculated as the ratio of the indirect effect to the total effect. Mediators were classified as positive (indirect effect > 0) or negative (indirect effect < 0) based on the direction of the estimated indirect effect.

### 2.6. Colocalization Analysis

To establish whether the relationships between gene expression, age-related hearing loss (ARHL), and Alzheimer’s disease (AD) could be attributed to shared causal variants, thus differentiating the confounding effects of linkage disequilibrium, we carried out a Bayesian colocalization analysis based on summary statistics from gene expression, ARHL, and AD genome-wide association studies (GWAS) [20]. Colocalization analyses were performed independently for each candidate mediating gene with ARHL and with the corresponding AD phenotype, respectively. Five hypotheses were involved in the colocalization analysis: (1) Neither gene expression nor the disease (ARHL/AD) had a causal variant in the genomic region (H0); (2) A causal variant was present solely in gene expression (H1); (3) A causal variant was present solely in the disease (ARHL/AD) (H2); (4) Distinct causal variants were present for gene expression and the disease (ARHL/AD) (H3); (5) A shared causal variant existed for gene expression and the disease (ARHL/AD) (H4). Posterior probabilities for each hypothesis were calculated to quantify the support for a shared genetic signal, and a posterior probability for hypothesis 4 (PP.H4) of ≥0.8 was considered strong evidence for a shared causal variant driving the observed associations between gene expression and the relevant disease phenotype (ARHL or AD), while a PP.H4 value between 0.5 and 0.8 indicated a moderate level of colocalization evidence [21]. For visualizing the regional results of colocalization analyses, we used the “LocusCompareR” package [22].

### 2.7. Replication Analysis

Genes prioritized based on mediation and colocalization evidence were further evaluated in replication analyses using independent eQTL data from the eQTLGen consortium. Mendelian randomization analyses were repeated following the same procedures as in the discovery stage. Directional consistency and statistical significance were assessed to determine reproducibility.

### 2.8. Protein-Protein Interaction Network Analysis

To explore potential functional interactions among mediating genes, protein-protein interaction analysis was conducted using the STRING database (https://string-db.org/, accessed on 1 January 2026) with the species restricted to Homo sapiens and default interaction parameters. Disconnected nodes were excluded. Interaction data were exported and visualized in Cytoscape (version 3.9.1). Network topology was analyzed using the Network Analyzer tool, and degree centrality was used to identify hub genes within the interaction network.

### 2.9. Functional Enrichment Analysis

Gene Ontology and Kyoto Encyclopedia of Genes and Genomes pathway enrichment analyses were performed to explore the biological functions and pathways associated with the identified mediating genes. Enrichment analyses were conducted using the “clusterProfiler” package [23] in R with standard overrepresentation methods, and false discovery rate correction was applied to account for multiple comparisons. Terms and pathways with adjusted *p* values below 0.05 were considered statistically significant.

## 3. Results

### 3.1. Overview of the Study Design

**Figure 1:**
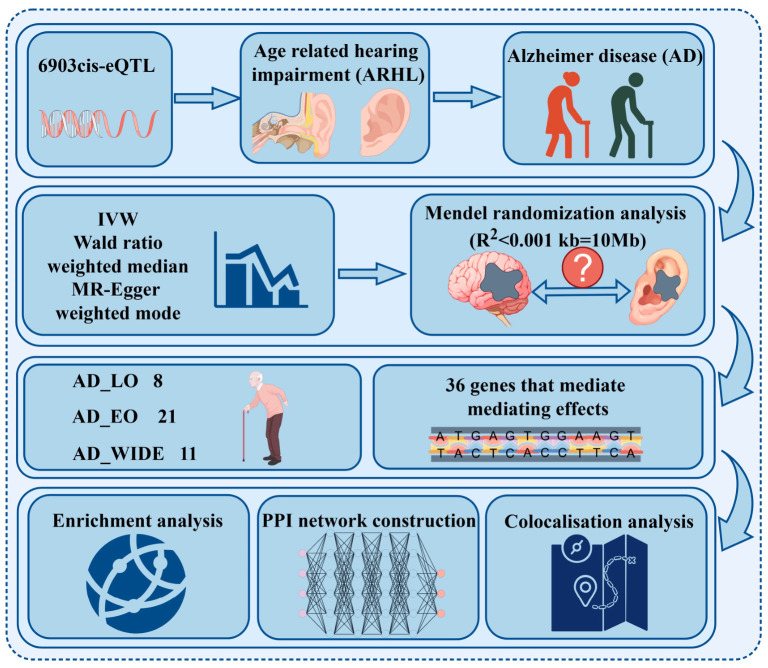
Flowchart of the study design. The workflow illustrates the sequential procedures, including Mendelian randomization analyses of ARHL and AD, reverse MR, cis-eQTL-based mediation analysis, Bayesian colocalization, replication validation, protein-protein interaction network construction, and functional enrichment analyses.

A schematic overview of the analytical framework is presented in Figure 1. In this study, we systematically investigated the genetic relationship between age-related hearing loss (ARHL) and Alzheimer’s disease (AD) through an integrative analytical strategy. Mendelian randomization analyses were first performed to evaluate the potential causal effect of ARHL on multiple AD phenotypes. Reverse Mendelian randomization analyses were additionally conducted to assess potential reverse causality between AD and ARHL, thereby strengthening the inference regarding directionality.

Building upon this framework, cis-eQTL-based analyses were subsequently implemented to identify genetically regulated genes that may mediate the association between ARHL and AD. Replication analyses using independent datasets were further performed to validate the robustness and consistency of the identified associations. To strengthen causal inference and prioritize reliable candidates, colocalization analysis was conducted to determine whether shared genetic variants underlie the observed associations. In addition, protein-protein interaction network construction and functional enrichment analyses were performed to characterize the biological relevance and functional context of the identified mediating genes. Collectively, this multi-layered analytical framework enabled a comprehensive evaluation of the genetic mechanisms linking ARHL and AD.

### 3.2. MR Estimates for the Association Between ARHL and AD

**Figure 2:**
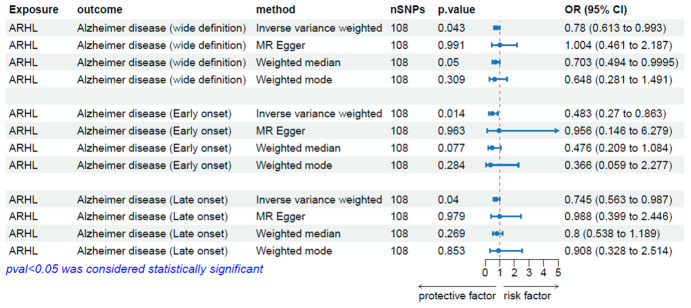
Forest plot of Mendelian randomization estimates for the association between age-related hearing loss and Alzheimer’s disease and its subtypes. Forest plot showing odds ratios (ORs) and 95% confidence intervals (CIs) for the association between genetically predicted age-related hearing loss (ARHL) and Alzheimer’s disease outcomes, including overall AD (AD_WIDE), early-onset AD (AD_EO), and late-onset AD (AD_LO). Effect estimates were derived using the inverse variance weighted (IVW) method as the primary MR approach. The vertical dashed line represents the null effect (OR = 1).

Using genetic instruments associated with age-related hearing loss at a significance threshold of *p* < 1 × 10^−5^, Mendelian randomization analyses were performed to evaluate its genetic association with Alzheimer’s disease and its subtypes. The effect estimates across Alzheimer’s disease outcomes, including overall AD and its subtypes, are illustrated in Figure 2.

Inverse variance weighted analysis indicated that genetically predicted ARHL was associated with a reduced risk of overall Alzheimer’s disease, with nominal statistical significance (IVW: β = −0.25, OR = 0.78, 95% CI: 0.63–0.99, *p* = 0.043).

In subtype analyses, ARHL showed a stronger inverse association with early-onset Alzheimer’s disease (IVW: β = −0.73, OR = 0.48, 95% CI: 0.27–0.86, *p* = 0.014). A modest but statistically significant association was also observed for late-onset AD (IVW: β = −0.29, OR = 0.75, 95% CI: 0.56–0.99, *p* = 0.040).

Leave-one-out sensitivity analyses showed that sequential removal of each genetic variant did not materially alter the pooled IVW estimates for any of the three AD outcomes (Supplementary Figures S1–S3), suggesting that the findings were not unduly influenced by any single SNP.

A comprehensive summary of MR diagnostic metrics, including F-statistics, Cochran’s Q statistics with *p*-values for heterogeneity, and MR-Egger intercepts with *p*-values for horizontal pleiotropy, is provided in Supplementary Table S8.

Across all analyses, no substantial heterogeneity or evidence of horizontal pleiotropy was detected, and Steiger directionality tests consistently supported the directionality from ARHL genetic liability to Alzheimer’s disease outcomes.

### 3.3. Reverse MR Analysis: Alzheimer’s Disease and Age-Related Hearing Loss

To further assess the directionality of the relationship between age-related hearing loss (ARHL) and Alzheimer’s disease, we conducted reverse Mendelian randomization analyses to evaluate whether Alzheimer’s disease could influence ARHL. Genetic instruments for Alzheimer’s disease, including overall AD (AD_WIDE), early-onset AD (AD_EO), and late-onset AD (AD_LO), were used in the analysis. The results from the reverse causal analysis indicated no significant effect of genetically predicted Alzheimer’s disease on ARHL in any of the tested phenotypes, with *p*-values of 0.688 for early-onset AD, 0.649 for late-onset AD, and 0.268 for overall AD. These findings suggest that the association between ARHL and Alzheimer’s disease is unlikely to be driven by reverse causality, supporting the primary hypothesis that ARHL may influence the risk of Alzheimer’s disease (Supplementary Table S3).

### 3.4. Identification of eQTLs with Mediating Effects Between ARHL and AD

**Figure 3:**
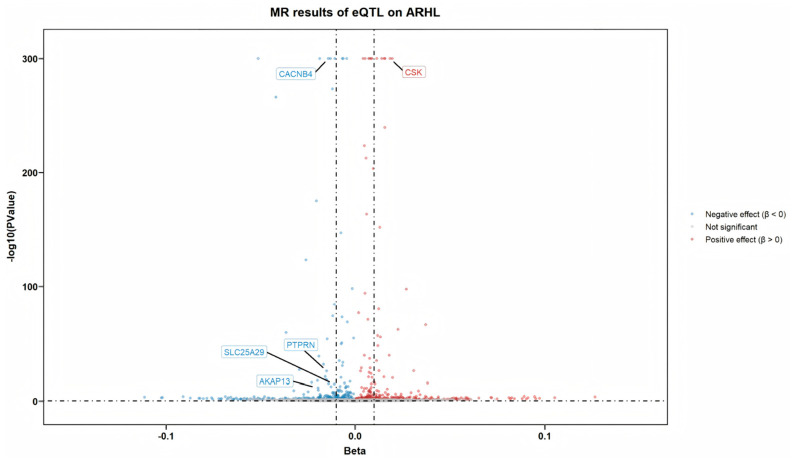
Volcano plot of cis-eQTL associations with age-related hearing loss. Volcano plot displaying the association results of cis-eQTLs with age-related hearing loss (ARHL). The x-axis represents effect sizes, and the y-axis represents −log10(*p* values). Red and blue dots indicate eQTLs that passed the false discovery rate (FDR) correction (FDR < 0.05), with red representing positive effect sizes (β > 0) and blue representing negative effect sizes (β < 0). Grey dots represent non-significant associations (FDR ≥ 0.05).

Subsequently, each of the 6903 cis-eQTLs was treated as a genetic exposure in separate Mendelian randomization analyses with ARHL and AD phenotypes as outcomes. Alzheimer’s disease outcomes included overall AD (AD_WIDE), early-onset AD (AD_EO), and late-onset AD (AD_LO). For each analysis, false discovery rate (FDR) correction was applied, and eQTLs with FDR < 0.05 were considered statistically significant. The corresponding FDR-adjusted *p*-values are provided in Supplementary Tables S4–S7. The distribution of cis-eQTL associations with ARHL is illustrated in the volcano plot (Figure 3), where significantly associated genes after FDR correction are highlighted.

**Figure 4:**
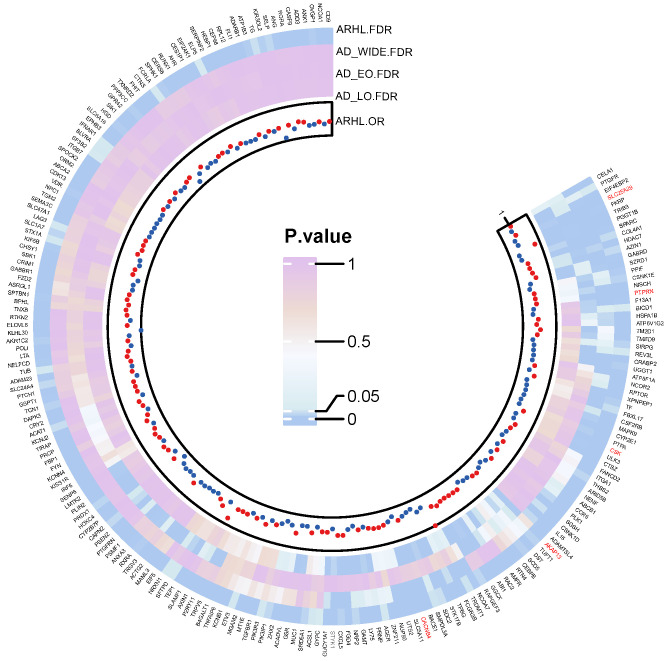
Circular heatmap summarizing cis-eQTL associations across age-related hearing loss and Alzheimer’s disease phenotypes. The circular heatmap summarizes cis-eQTLs significantly associated with age-related hearing loss (ARHL) and Alzheimer’s disease phenotypes, including overall AD (AD_WIDE), early-onset AD (AD_EO), and late-onset AD (AD_LO), with all displayed associations passing FDR correction (FDR < 0.05). The heatmap also displays odds ratios (ORs) for ARHL. Color intensity represents the magnitude and direction of effect sizes, with red indicating positive effects and blue indicating negative effects.

Through comparative statistical screening, we identified eQTLs that were significantly associated with ARHL and concurrently associated with at least one AD phenotype. The overall distribution and overlap of significant eQTLs across ARHL and AD phenotypes are summarized in a circular heatmap (Figure 4), illustrating the cross-phenotype significance patterns among AD_WIDE, AD_EO, and AD_LO. These overlapping eQTLs were then subjected to formal mediation analysis to evaluate whether their effects on AD outcomes were mediated through ARHL.

Based on the direction and magnitude of the estimated indirect effects (IE), mediating eQTLs were classified into positive mediators (IE > 0) and negative mediators (IE < 0). Positive mediating effects were observed for *PTPRN*, *CACNB4*, *ATP6V1G2*, *CELA1*, and *FCGR3B* in AD_WIDE; *SLC25A29*, *PTPRN*, *AKAP13*, *AXIN1*, *CSNK1E*, *LITK2*, *MAML3*, *REV3L*, *TEP1*, and *TIED9* in AD_EO; and *SLC25A29*, *CACNB4*, *AKAP13*, *GGCX*, *AMFR*, and *GAMT* in AD_LO.

Conversely, negative mediating effects were identified for *ADAMTSL4*, *ATP5F1A*, *CSK*, *HSPA1B*, *NUP50*, and *ULK3* in AD_WIDE; *FKRP*, *PGGT1B*, *UGGT1*, *ABI1*, *EIF5*, *P2RY11*, *PLIN2*, *PPIF*, *PVR*, *RAC2*, and *TRPV5* in AD_EO; and *ACP5* and *RTN4* in AD_LO.

Finally, we examined the consistency of mediating effects across AD phenotypes. Among the identified mediating eQTLs, four genes demonstrated concordant positive mediating effects across two AD subtypes, with statistically significant indirect effects supported by Sobel tests. Specifically, *PTPRN* showed a consistent mediating role in both AD_WIDE and AD_EO, accounting for 6.57% of the ARHL-AD_WIDE association (Sobel *p* = 0.047) and 7.40% of the ARHL-AD_EO association (Sobel *p* = 0.017), respectively. *CACNB4* exhibited mediating effects in AD_WIDE and AD_LO, with mediation proportions of 4.36% (*p* = 0.043) and 2.11% (*p* = 0.040).

In addition, *SLC25A29* demonstrated consistent mediation in AD_EO and AD_LO, explaining 15.47% (*p* = 0.019) and 6.30% (*p* = 0.047) of the respective ARHL-AD associations. Notably, *AKAP13* showed the strongest mediating effect among shared eQTLs, mediating 26.26% of the ARHL-AD_EO association (*p* = 0.021) and 9.62% of the ARHL-AD_LO association (*p* = 0.049). No eQTL exhibited consistent mediating effects across all three AD phenotypes.

In contrast, eQTLs with negative mediating effects were largely phenotype-specific, and no negatively mediating eQTL was shared by two or more AD phenotypes.

### 3.5. Colocalization Analysis of Mediating eQTLs

To further validate whether the observed mediating effects were driven by shared causal variants rather than linkage disequilibrium, we performed Bayesian colocalization analyses for all eQTLs identified in the mediation analysis. Colocalization was conducted separately between each candidate eQTL and age-related hearing loss (ARHL), as well as between the same eQTL and its corresponding Alzheimer’s disease (AD) phenotype.

**Figure 5:**
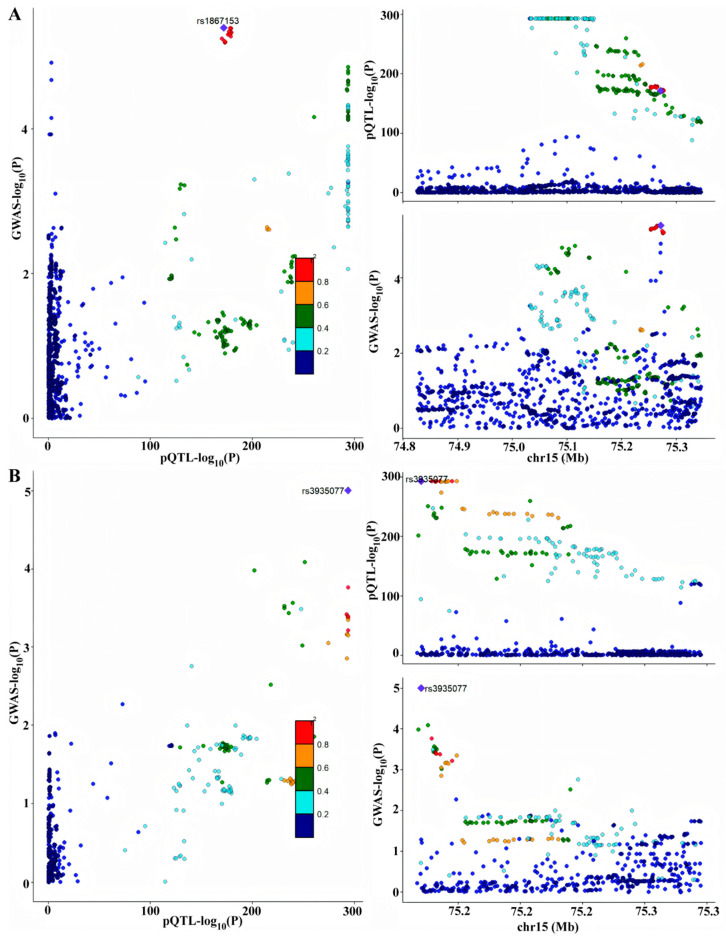
Bayesian colocalization analysis of the *CSK* locus with age-related hearing loss and Alzheimer’s disease. Regional association plots showing colocalization results between the *CSK* cis-eQTL and (**A**) age-related hearing loss (ARHL) and (**B**) overall Alzheimer’s disease (AD_WIDE). Each dot represents a genetic variant within the locus. The x-axis indicates genomic position, and the y-axis shows −log10(*p* values). Colors indicate linkage disequilibrium with the lead variant. High posterior probability for hypothesis 4 (PP.H4) supports the presence of a shared causal variant underlying both traits.

Among all tested mediating eQTLs, only *CSK* demonstrated strong evidence of colocalization with both ARHL and AD outcomes. Specifically, the *CSK* locus showed a high posterior probability for a shared causal variant with ARHL (PP.H4 = 0.832) and an even stronger colocalization signal with overall Alzheimer’s disease (AD_WIDE; PP.H4 = 0.991). The regional association plots further illustrated a consistent overlap of association signals between *CSK* expression and both ARHL and AD_WIDE within the same genomic locus (Figure 5A,B). These results indicate that the associations of *CSK* with ARHL and AD_WIDE are likely driven by the same underlying genetic variant.

In contrast, none of the remaining mediating eQTLs exhibited robust colocalization signals (PP.H4 > 0.8) simultaneously with ARHL and their corresponding AD phenotypes, despite showing statistically significant indirect effects in the mediation analysis. This suggests that, for these loci, the observed mediation effects may be attributable to distinct causal variants in linkage disequilibrium or more complex genetic architectures rather than a single shared causal signal. Based on this combined evidence, the mediating genes identified in this study were categorized into two tiers: Tier 1 (high confidence)—CSK, supported by both significant mediation and robust colocalization with both ARHL and AD_WIDE, and Tier 2 (suggestive)—the remaining 35 genes, which showed statistical mediation signals but lacked colocalization support and should therefore be interpreted with caution.

### 3.6. Replication Analysis of Prioritized Mediating eQTLs

Based on the mediation and colocalization analyses, five eQTLs were prioritized for replication to further evaluate the robustness of the identified mediating signals. Replication analyses were conducted using an independent eQTL dataset from the eQTLGen consortium.

Among the five prioritized eQTLs, *CSK* and *PTPRN* showed statistically significant and directionally consistent associations with age-related hearing loss, supporting the stability of their regulatory effects on the intermediate phenotype.

We next examined whether the originally observed mediation effects on Alzheimer’s disease outcomes could be replicated. For *CSK*, the association with overall Alzheimer’s disease (AD_WIDE) remained statistically significant in the replication dataset. Mediation analysis indicated that *CSK* continued to exert an indirect effect on AD_WIDE through ARHL, with a mediation proportion of 3.58%, comparable to that observed in the discovery analysis. Although the Sobel test for the indirect effect did not reach conventional statistical significance (Sobel *p* = 0.078), the direction and magnitude of the mediation effect were consistent, suggesting a stable mediating role of *CSK*.

In contrast, although *PTPRN* replicated at the eQTL-ARHL level, its association with AD outcomes did not reach statistical significance in the replication analysis (*p* > 0.05), and mediation analysis was therefore not pursued.

To further explore brain-specific regulatory signals, we additionally screened for cis-eQTLs of the five prioritized genes (*CSK*, *AKAP13*, *PTPRN*, *SLC25A29*, *CACNB4*) in two AD-relevant brain regions from GTEx v8: Brain-Hippocampus and Brain-Frontal Cortex. No valid cis-eQTLs were detected for any of the five genes in either brain region, even after relaxing the significance threshold to *p* < 1 × 10^−5^.

Taken together, these results indicate that among the five prioritized mediating eQTLs, CSK showed the most consistent directional evidence across discovery and replication analyses, although the replication was partial as the Sobel test did not reach statistical significance (Sobel *p* = 0.078), likely due to reduced statistical power or differences in tissue composition between datasets.

### 3.7. Protein-Protein Interaction (PPI) Network Analysis

To further explore the potential functional interactions among genes identified in the mediation analysis, protein-protein interaction (PPI) network analysis was performed. A total of 36 mediating genes were included in the PPI network construction.

**Figure 6:**
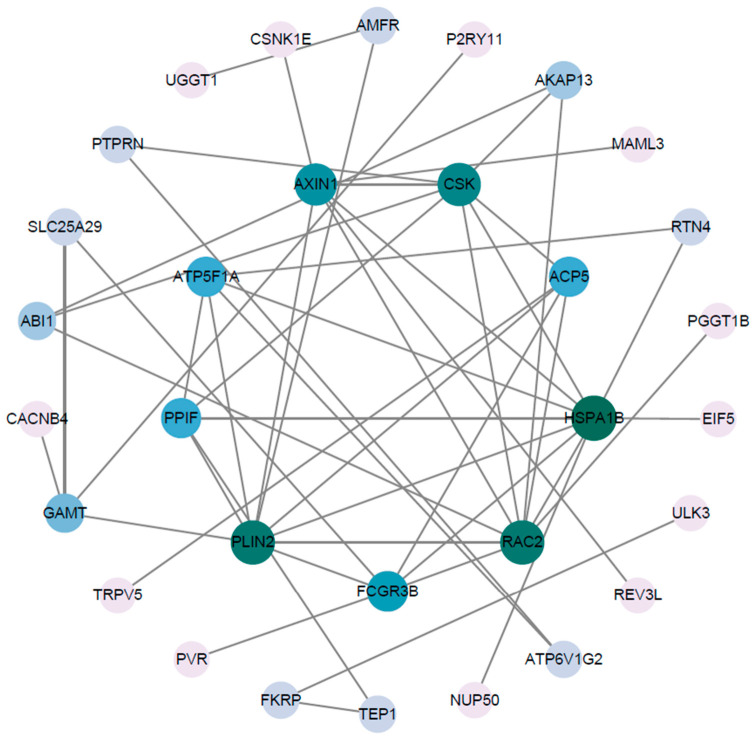
Protein-protein interaction (PPI) network of common targets. PPI network diagram of common targets. In the network, darker node colors and larger circle sizes indicate greater importance of the key targets, while the edges represent the interactions between these targets.

The PPI visualization results (Figure 6) demonstrated that several key targets, including *CSK*, *AKAP13*, *PTPRN*, *SLC25A29*, and *CACNB4*, formed interconnected interaction clusters and showed relatively dense interaction patterns within the network. Table 2 summarizes the basic information of the key targets ranked according to their network connectivity, where higher-ranking genes indicate stronger interaction connectivity and potentially more central roles within the PPI network.

### 3.8. GO and KEGG Enrichment Analysis

To further explore the potential biological functions and pathways underlying the mediating effects between age-related hearing loss (ARHL) and Alzheimer’s disease (AD), Gene Ontology (GO) and Kyoto Encyclopedia of Genes and Genomes (KEGG) enrichment analyses were performed. A total of 36 genes identified from the mediation analysis were included in the enrichment analysis.

**Figure 7:**
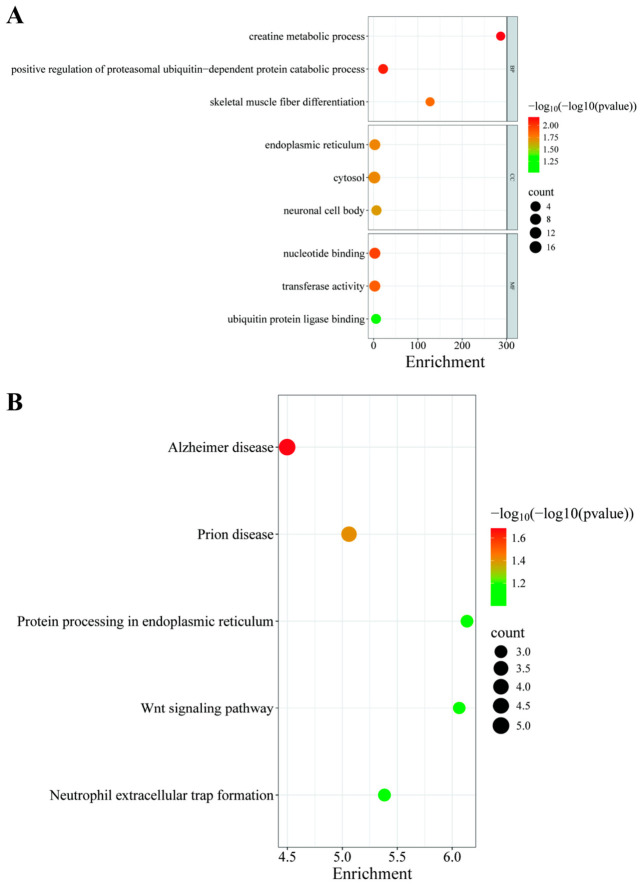
GO and KEGG enrichment analyses. (**A**) Bubble plot of Gene Ontology (GO) enrichment analysis. GO terms were ranked according to false discovery rate (FDR) values from lowest to highest, and the top three enriched terms from each GO category (biological process, cellular component, and molecular function) were selected for visualization in a combined bubble plot. The size of each bubble represents the number of enriched genes (gene counts), with larger bubbles indicating a greater number of target genes involved in the corresponding term. The color intensity of the bubbles reflects the enrichment significance, with darker colors indicating higher enrichment significance. (**B**) Bubble plot of Kyoto Encyclopedia of Genes and Genomes (KEGG) pathway enrichment analysis. Pathways were ranked based on FDR values, and the top five enriched pathways were visualized. Larger bubble sizes and darker colors indicate higher enrichment levels and greater statistical significance of the corresponding pathways.

GO enrichment analysis demonstrated that the prioritized genes were significantly enriched in biological processes related to the creatine metabolic process and protein catabolic processes. In the cellular component category, enriched terms were mainly associated with the endoplasmic reticulum, cytosol, and neuronal cell body. Molecular function analysis indicated significant enrichment in nucleotide binding and transferase activity (Figure 7A).

KEGG pathway enrichment analysis further showed that these genes were primarily involved in pathways related to Alzheimer’s disease, Wnt signaling, and protein processing in the endoplasmic reticulum (Figure 7B), suggesting potential functional relevance to neurodegenerative processes.

## 4. Discussion

In the present study, we systematically investigated the genetic relationship between age-related hearing loss (ARHL) and Alzheimer’s disease (AD) using an integrative genomic framework including Mendelian randomization (MR), cis-eQTL analysis, mediation, Bayesian colocalization, replication, and systems-level functional analyses. We found that genetically predicted ARHL was associated with reduced risk of AD and its subtypes, with no reverse causal effect of AD on ARHL. We further identified multiple genes that may mediate the ARHL-AD association, among which CSK showed robust colocalization and replication as a shared causal locus. PPI and enrichment analyses revealed that these mediators are involved in neurodegeneration, cellular metabolism, and protein processing pathways. Collectively, our findings provide preliminary genetic evidence for a shared regulatory architecture, although the observed direction warrants further investigation between ARHL and AD.

A substantial body of epidemiological evidence has reported that hearing loss is associated with an increased risk of cognitive decline and dementia [3,24,25,26]. For example, large longitudinal cohort studies have shown that individuals with hearing impairment exhibit a significantly higher risk of developing dementia compared with those with normal hearing, with risk increasing according to the severity of hearing loss [3,24]. Furthermore, hearing loss has been identified as one of the most important potentially modifiable risk factors for dementia in the Lancet Commission on dementia prevention [5]. However, the inverse association between genetically predicted ARHL and AD risk observed in our MR analyses appears to contradict this well-established epidemiological evidence. Several factors may explain this discrepancy. First, Mendelian randomization estimates reflect the cumulative effect of lifelong genetic susceptibility to ARHL, rather than the acquired, late-life sensorineural hearing loss captured in observational studies [27,28]. Genetic variants influencing ARHL may also affect neurodevelopmental trajectories, synaptic plasticity, or immune responses in ways that coincidentally correlate with reduced AD risk through antagonistic pleiotropy—a phenomenon where the same genetic variant exerts opposite effects on different phenotypes at different life stages. Second, the ARHL GWAS used in this study inevitably includes variants with pleiotropic effects on general neurodegeneration, cognitive reserve, or systemic inflammation [29]. These pleiotropic pathways may contribute to the observed negative estimates without implying that clinical hearing loss itself is protective. Third, the modest effect sizes and borderline *p*-values (e.g., OR = 0.78, *p* = 0.043 for AD_WIDE) underscore that our findings are statistically fragile and should be interpreted as hypothesis-generating rather than confirmatory. Therefore, we emphasize that our results should not be clinically interpreted as “hearing loss preventing AD” but rather as a genetic epidemiological signal that warrants further mechanistic dissection, particularly at the level of CSK-mediated transcriptional regulation. Notably, some observational studies have also suggested that hearing impairment may represent an early manifestation of neurodegenerative processes rather than a causal factor contributing to cognitive decline [30,31], which further supports the notion that the relationship between ARHL and AD is complex and bidirectional.

In addition to these general considerations, specific forms of bias inherent to GWAS and MR should be considered as alternative explanations for the unexpected direction. Horizontal pleiotropy, where genetic instruments affect AD through pathways independent of ARHL, remains a concern despite our sensitivity analyses showing no evidence of pleiotropy (MR-Egger intercept *p* > 0.05 for all analyses). Similarly, survival bias may influence the ARHL GWAS, as individuals with severe hearing impairment or cognitive decline are less likely to be enrolled in population-based cohorts, potentially distorting allele frequency estimates. Dynastic effects (i.e., parental genotypes influencing offspring phenotypes via environmental pathways rather than inherited alleles) could also contribute to the observed inverse association, although MR methods are generally robust to such biases when using cis-eQTLs as instruments. While we cannot definitively exclude these possibilities, the consistency of our findings across multiple sensitivity analyses and the robust colocalization evidence for CSK argue against these biases being the sole drivers of our results.

If the genetic association reflects a genuine biological link rather than bias, what mechanism might account for a protective direction? We hypothesize a CSK-centered neuroimmune resilience pathway. CSK encodes C-terminal Src kinase, a negative regulator of Src family kinases (SFKs), including Fyn, which plays a critical role in amyloid-β-induced synaptic dysfunction and tau phosphorylation in AD [32,33]. Genetically upregulated CSK expression may dampen SFK activity, thereby reducing neuroinflammatory responses and neuronal damage in the aging brain, potentially lowering AD risk. Conversely, the same genetic variant in the auditory periphery may predispose to cochlear degeneration through distinct cell-type-specific mechanisms (e.g., impaired hair cell survival or synaptic maintenance), representing a classic example of antagonistic pleiotropy—a single genetic locus exerting opposite effects on different tissues or at different life stages. This hypothesis is speculative and requires functional validation in relevant cellular and animal models, but it provides a testable framework for future mechanistic studies.

Beyond causal inference, our integrative analyses aimed to identify transcriptional regulatory mechanisms that may connect ARHL and AD. By incorporating cis-eQTL data, we identified multiple genes whose genetically regulated expression was associated with both ARHL and AD outcomes. Several genes demonstrated consistent mediation signals across AD phenotypes, including *PTPRN*, *CACNB4*, *SLC25A29*, and *AKAP13*. However, unlike CSK, these loci lacked robust colocalization evidence (PP.H4 < 0.8); therefore, their mediating roles should be considered suggestive rather than confirmed. These genes have previously been implicated in neuronal signaling, synaptic function, and cellular metabolism, suggesting plausible biological mechanisms linking auditory dysfunction with neurodegenerative processes [34]. For example, *CACNB4* encodes a β subunit of voltage-gated calcium channels that regulate neuronal excitability and synaptic transmission. Calcium signaling plays a critical role in neuronal function, and dysregulation of calcium homeostasis has been implicated in both cochlear degeneration and Alzheimer’s disease pathology [35,36]. Similarly, *AKAP13* belongs to the A-kinase anchoring protein family and functions as a scaffolding protein that coordinates intracellular signaling pathways [37], including those involved in cytoskeletal organization and cell survival. Alterations in these signaling pathways may influence both auditory neuron integrity and neuronal vulnerability in the central nervous system.

Among all mediating genes identified in the present study, *CSK* demonstrated the most robust evidence supporting a shared genetic mechanism between ARHL and AD, although this evidence remains correlational and warrants further validation. Bayesian colocalization analysis indicated a high posterior probability for a shared causal variant between *CSK* expression and both ARHL and AD_WIDE, suggesting that the observed associations may arise from the same underlying genetic locus. Replication analyses using independent eQTL data from the eQTLGen consortium showed directionally consistent associations, further supporting the reproducibility of this finding, although the mediation effect was statistically partial. *CSK* encodes C-terminal Src kinase, a key negative regulator of Src family kinases that modulate multiple cellular signaling pathways [38]. Src family kinases have been implicated in neuronal development, synaptic plasticity, and neuroinflammatory signaling [39]. Dysregulation of Src-related pathways has also been associated with amyloid-β-induced synaptic dysfunction and neuronal damage in Alzheimer’s disease [32,33]. Therefore, genetic regulation of *CSK* expression may influence neuronal signaling networks that affect both cochlear function and neurodegenerative processes. Although the precise role of *CSK* in ARHL and AD remains to be elucidated, our findings suggest that this gene may represent a potential molecular link between auditory dysfunction and neurodegeneration.

The systems-level analyses performed in this study further support the biological relevance of the identified mediating genes. PPI network analysis revealed that CSK occupied the most central position within the interaction network (degree centrality = 8), while other prioritized genes including AKAP13 (degree = 3), PTPRN (degree = 2), SLC25A29 (degree = 2), and CACNB4 (degree = 1) showed more modest connectivity, suggesting potential functional coordination but with CSK as the primary hub. Functional enrichment analyses indicated that these genes were significantly enriched in pathways related to metabolic regulation, protein catabolism, and neuronal cellular components [40,41]. Notably, KEGG pathway analysis highlighted enrichment in pathways associated with Alzheimer’s disease, Wnt signaling, and protein processing in the endoplasmic reticulum [42,43]. These pathways have been widely implicated in neurodegenerative disorders. For instance, endoplasmic reticulum stress and impaired protein processing contribute to the accumulation of misfolded proteins and neuronal dysfunction in Alzheimer’s disease [44,45]. Similarly, Wnt signaling plays important roles in synaptic maintenance and neuronal survival, and dysregulation of this pathway has been implicated in AD pathogenesis [42]. The enrichment of these pathways in our analysis suggests that shared molecular mechanisms may underlie both auditory system degeneration and neurodegenerative processes in the brain [46,47].

This study has several key strengths. First, integrating Mendelian randomization and cis-eQTL analyses enabled a multi-layered assessment of causal pathways linking ARHL and AD. Second, Bayesian colocalization helped distinguish genuine shared causal variants from LD-driven confounding. Third, independent replication datasets enhanced result robustness. Finally, PPI and functional enrichment analyses provided biological context for the identified genes and pathways.

### 4.1. Limitations of the Study

Several limitations should be noted when interpreting our results. First, all GWAS, eQTL, and replication datasets were limited to European ancestry, restricting generalizability to other populations. Population-specific genetic architecture, LD patterns, and environmental interactions may alter the ARHL-AD association and regulatory effects of identified mediators [48].

Second, our cis-eQTL analyses were mainly based on blood-derived data, not auditory or brain tissues critical to ARHL and AD pathogenesis. Peripheral blood eQTLs may miss tissue- or cell-type-specific regulatory mechanisms driving the ARHL-AD link [49]. We detected no valid cis-eQTLs for the five prioritized genes in hippocampal or frontal cortical tissues, likely due to small sample sizes and tissue-specific regulatory divergence. This absence of brain eQTL signals, while methodologically limiting, may also reflect genuine biological differences between peripheral and central compartments—suggesting that the regulatory effects captured in blood do not simply mirror those in the brain, and that the ARHL-AD link may involve systemic (e.g., immune-mediated) pathways rather than local brain transcriptional changes alone. Consequently, our mediation results, particularly those for genes lacking brain eQTL support, should be interpreted as reflecting genetically regulated expression in the peripheral compartment, and their extrapolation to inner ear or brain mechanisms remains speculative. Future studies using tissue-specific eQTL data from cochlear and brain tissues are urgently needed to determine whether the mediating genes identified here exert similar regulatory effects in the target organs relevant to ARHL and AD pathogenesis.

Third, despite FDR correction, the large number of cis-eQTLs tested (n = 6903) carries a risk of false positives. Only CSK showed strong colocalization with both ARHL and AD; other mediators lacked robust evidence (PP.H4 < 0.8), suggesting possible LD or complex architecture confounding [50].

Fourth, our cis-eQTL analysis was restricted to genes annotated as ‘druggable’ based on the Finan et al. druggable genome and DGIdb. This focus was intentional, as it prioritizes targets with potential for therapeutic translation and aligns with established practices in systematic druggable genome-wide MR studies. However, we acknowledge that this selection strategy may have introduced bias by excluding non-druggable genes that could also play important mediating roles in the ARHL-AD relationship. Thus, while our approach is hypothesis-generating for drug development, the identified mediators represent only a subset of the broader biological landscape.

Fifth, the MR analyses for the causal effect of ARHL on AD used a *p*-value threshold of 1 × 10^−5^ for instrument selection, which is less stringent than the conventional genome-wide significance threshold of 5 × 10^−8^. This threshold was chosen to retain a sufficient number of genetic instruments for the ARHL exposure GWAS, as the number of genome-wide significant SNPs was limited. Importantly, this threshold has been adopted in previous large-scale MR studies and is considered acceptable when the exposure GWAS has moderate power. Nevertheless, we acknowledge that a more lenient threshold may increase the risk of including weak instruments or false-positive associations. To mitigate this concern, we calculated F-statistics for all instruments (all > 10, indicating sufficient instrument strength) and performed multiple sensitivity analyses (weighted median, MR-Egger, leave-one-out) that consistently supported the primary IVW findings. However, this limitation should be considered when interpreting the results.

Finally, all findings rely on in silico inference from summary genomic data without functional experimental validation. This prevents confirmation of direct causal links between candidate genes and ARHL/AD pathogenesis or clarification of their exact mediating mechanisms.

### 4.2. Future Perspectives

Future studies addressing these limitations will help refine and validate the genetic mechanisms linking ARHL and AD. First, cross-ancestry validation using non-European GWAS and eQTL data is urgently needed to evaluate universality and identify population-specific variants, thereby improving generalizability [48].

Second, tissue-specific eQTL analyses in the inner ear and key brain regions (hippocampus, temporal cortex) should be conducted to detect cell-type-specific regulatory signals and refine core mediators of the ARHL-AD axis [9,49].

Third, methodological improvements will strengthen causal inference, including stricter colocalization thresholds, fine-mapping strategies to resolve LD-confounded signals [50,51], and multi-omics integration to build a complete regulatory network for mediating genes.

Fourth, functional experimental validation is critical to confirm the causal role of the top candidate genes. Gain- or loss-of-function studies using cellular models (e.g., cochlear hair cells, hippocampal neurons) and animal models (e.g., ARHL/AD double knockout mice) can be performed to examine the effects of these genes on auditory and cognitive phenotypes, as well as on key pathological processes (e.g., synaptic dysfunction, neuroinflammation, protein misfolding). For *CSK*, in particular, investigating its regulatory effects on Src family kinase signaling—a key pathway implicated in both ARHL and AD—will provide direct mechanistic evidence [32,38].

Finally, translating these genetic findings into clinical practice requires validation in real-world longitudinal clinical cohorts. Assessing the relationship between the expression levels of candidate genes, objective hearing thresholds, and cognitive decline in prospective cohorts will help determine the clinical relevance of our genetic findings and identify potential predictive biomarkers or therapeutic targets for the simultaneous intervention of ARHL and AD [5].

In conclusion, this study identifies a prioritized gene (CSK) that may serve as a molecular link for future mechanistic studies, while the overall ARHL-AD directionality should be interpreted with caution given the modest effect sizes and the counterintuitive direction. Using an integrative genomic framework combining Mendelian randomization, eQTL mediation, colocalization, replication, and functional analyses, we identified multiple genes that may mediate the ARHL-AD association, with CSK exhibiting the most consistent evidence among the prioritized genes across discovery and replication analyses, although the replication was statistically partial and warrants further validation in independent cohorts. These hypothesis-generating findings highlight transcriptional regulatory pathways that link auditory impairment to neurodegeneration and offer key directions for future mechanistic research and potential target prioritization, rather than immediate clinical or therapeutic applicability.

## Figures and Tables

**Table 1:** Summary of data sources and analytical applications.

Data Type	Data Source/Consortium	Ancestry	Sample Size (N)	Specific Application(s) in This Study
ARHL GWAS	EMBL-EBI (2020 meta-analysis)	European	330,759	• Primary exposure in forward MR • Outcome in reverse MR • cis-eQTL mediation analysis (exposure) • Bayesian colocalization with eQTLs
AD GWAS (Overall/EO/LO)	FinnGen (R12 release)	European	AD_WIDE: 496,753 AD_EO:218,292 AD_LO: 226,162	• Primary outcomes in forward MR • Exposure in reverse MR • cis-eQTL mediation analysis (outcomes) • Bayesian colocalization with eQTLs
Discovery cis-eQTLs	GTEx v8 (Whole Blood)	European	~838 donors	• Instrumental variables for mediating genes • Mediation analysis (Sobel test) • Bayesian colocalization with ARHL/AD GWAS
Replication cis-eQTLs	eQTLGen Consortium	European	31,684 individuals	• Independent replication of prioritized eQTL-mediated effects
Tissue-specific cis-eQTLs	GTEx v8 (Brain-Hippocampus & Frontal Cortex)	European	~225–250 donors each	• Exploratory validation of brain-specific regulatory signals for prioritized genes

Brain tissue sample sizes vary slightly across GTEx v8 releases and public repositories; the range reflects the reported numbers for hippocampus (N ≈ 165–255) and frontal cortex (N ≈ 175–269) across different sources.

**Table 2:** Topological Analysis of key targets.

Hub Gene	Average Shortest Path Length	Betweenness Centrality	Closeness Centrality	Degree
*CSK*	2.17	0.15	0.46	8
*AKAP13*	2.73	0	0.366	3
*PTPRN*	3.03	0.014	0.33	2
*SLC25A29*	3.03	0.0069	0.33	2
*CACNB4*	3.57	0	0.28	1

## Data Availability

The datasets analyzed during the current study are available in the following public repositories: summary-level genome-wide association study (GWAS) data for age-related hearing loss (ARHL) were obtained from the European Molecular Biology Laboratory-European Bioinformatics Institute (EMBL-EBI, https://www.ebi.ac.uk/, accessed on 1 January 2026); Alzheimer’s disease (AD) GWAS summary statistics, including overall AD, early-onset AD, and late-onset AD, were sourced from the FinnGen R12 release (https://www.finngen.fi/en/access_results, accessed on 1 January 2026); and cis-expression quantitative trait loci (cis-eQTL) data were obtained from the Genotype-Tissue Expression (GTEx) database (v8, https://www.gtexportal.org/home/, accessed on 1 January 2026) and the eQTLGen consortium (https://www.eqtlgen.org/cis-eqtls.html, accessed on 1 January 2026). Protein-protein interaction data were derived from the STRING database (https://string-db.org/, accessed on 1 January 2026). All data integration and statistical analyses were performed using R (version 4.4.1). Detailed results and foundational details of the datasets are provided within the article and its additional files.

## Supplementary Materials

The following supporting information can be downloaded at: https://www.mdpi.com/article/10.3390/genes17070776/s1. Supplementary Figure S1: Leave-one-out-ARHL-AD_EO. Supplementary Figure S2: Leave-one-out-ARHL-AD_LO. Supplementary Figure S3: Leave-one-out-ARHL-AD_WIDE. Table S1: outcome. Table S2: exposure eQTL. Table S3: reverse analysis. Table S4: eqtl-ARHL. Table S5: eqtl-AD_WIDE. Table S6: eqtl-AD_EO. Table S7: eqtl-AD_LO. Supplementary Table S8. MR diagnostic metrics for forward (ARHL → AD) and reverse (AD → ARHL) Mendelian randomization analyses.
