# Supplementary material for: The Genetic and Transcriptomic Nexus of Age-Related Hearing Loss and Alzheimer’s Disease
Source: Genes (Basel). 2026 Jun 30;17(7):776. doi: 10.3390/genes17070776 (PMC13409600; doi:10.3390/genes17070776)
Supplement: Supplementary file 1 [file genes-17-00776-s001.zip › Supplementary Figure S1_Leave-one-out-ARHL-AD_EO.pdf]

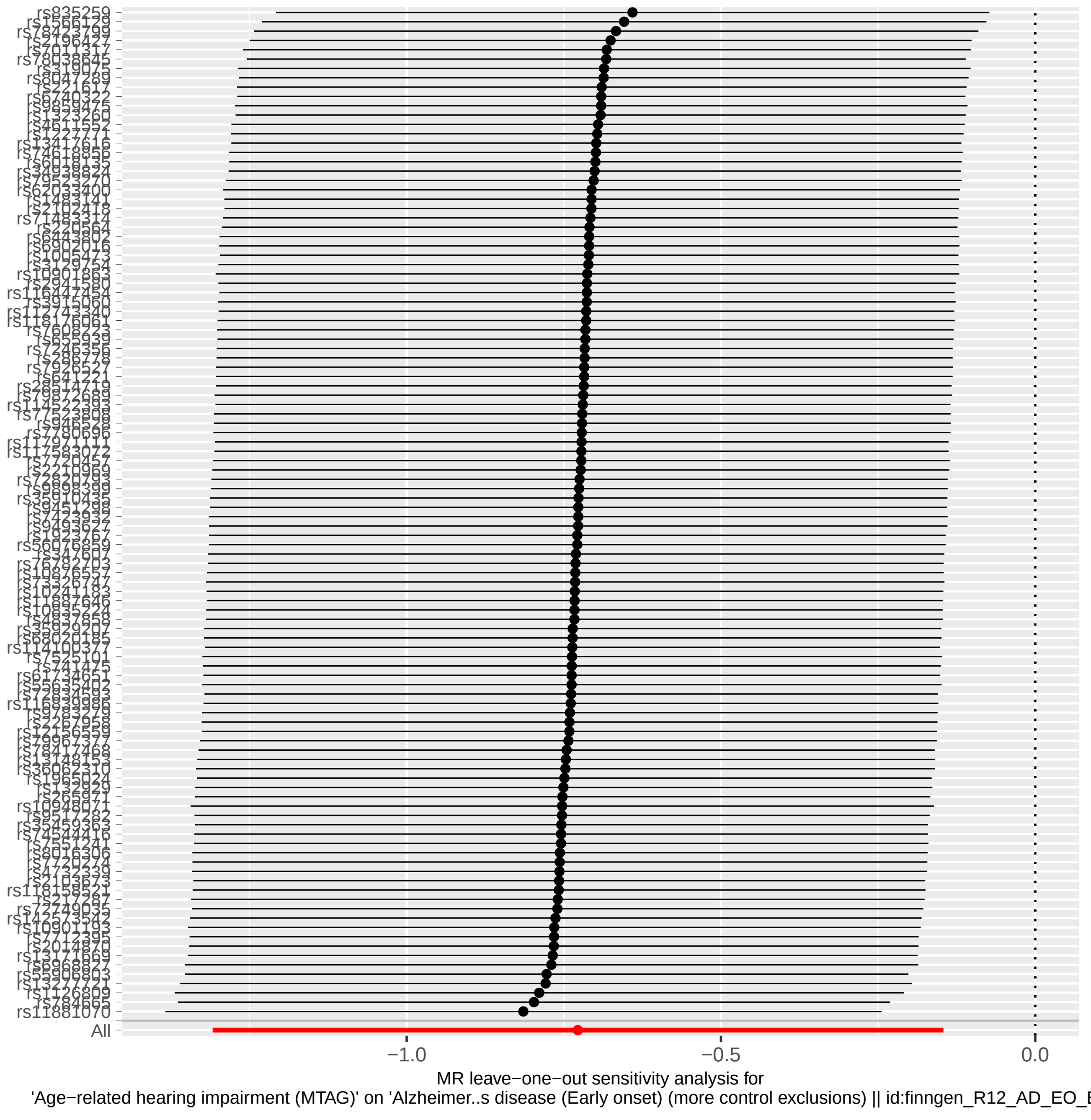
rs835259
rs11881070
All
−1.0
−0.5
0.0
MR leave−one−out sensitivity analysis for
'Age−related hearing impairment (MTAG)' on 'Alzheimer..s disease (Early onset) (more control exclusions) || id:finngen_R12_AD_EO_E
