# Supplementary material for: The Genetic and Transcriptomic Nexus of Age-Related Hearing Loss and Alzheimer’s Disease
Source: Genes (Basel). 2026 Jun 30;17(7):776. doi: 10.3390/genes17070776 (PMC13409600; doi:10.3390/genes17070776)
Supplement: Supplementary file 1 [file genes-17-00776-s001.zip › Supplementary Figure S2_Leave-one-out-ARHL-AD_LO.pdf]

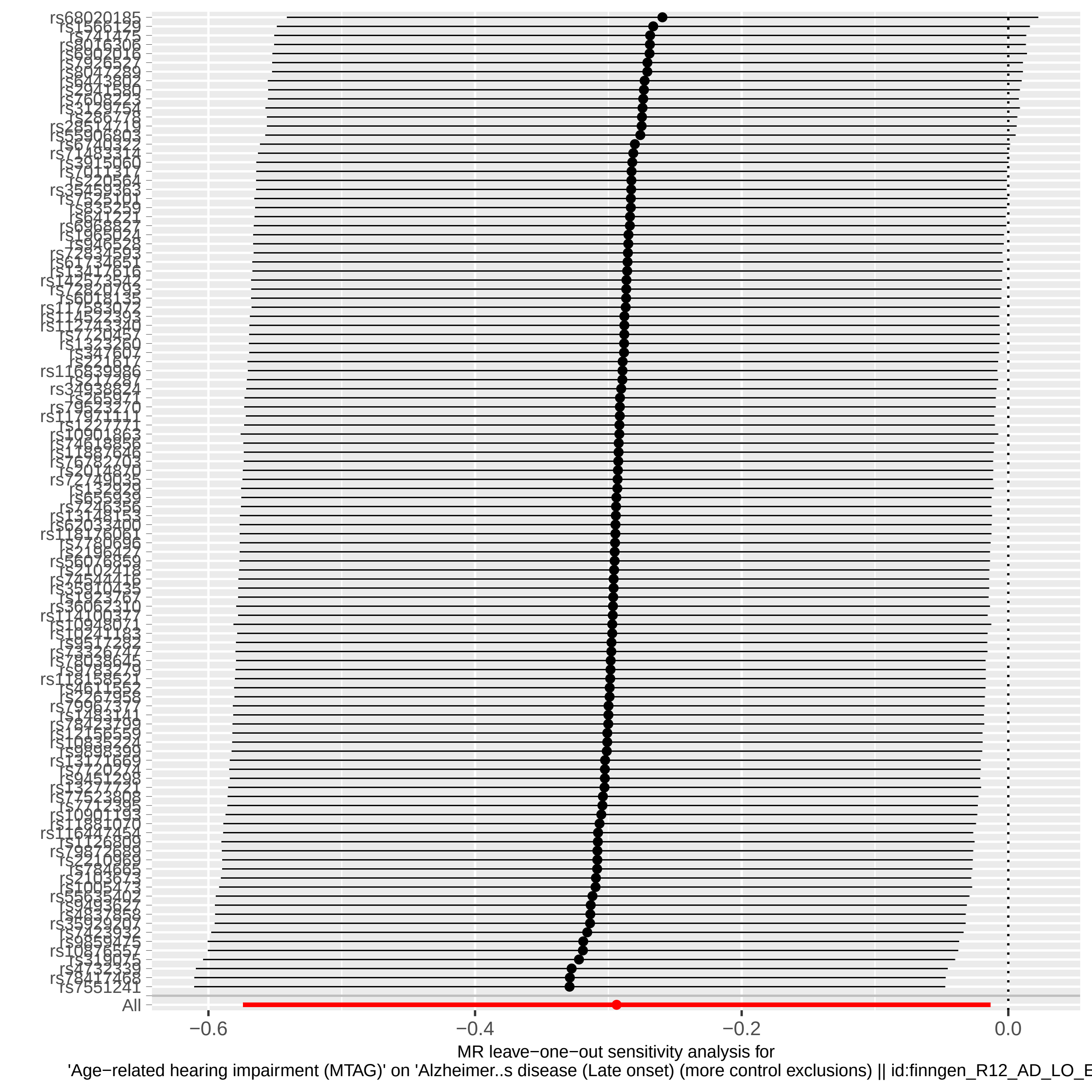
rs68020185
rs7551241
All
−0.6
−0.4
−0.2
0.0
MR leave−one−out sensitivity analysis for
'Age−related hearing impairment (MTAG)' on 'Alzheimer..s disease (Late onset) (more control exclusions) || id:finngen_R12_AD_LO_E
