# Supplementary material for: The Genetic and Transcriptomic Nexus of Age-Related Hearing Loss and Alzheimer’s Disease
Source: Genes (Basel). 2026 Jun 30;17(7):776. doi: 10.3390/genes17070776 (PMC13409600; doi:10.3390/genes17070776)
Supplement: Supplementary file 1 [file Supplementary Figure S3_Leave-one-out-ARHL-AD_WIDE.pdf › Supplementary Figure S3_Leave-one-out-ARHL-AD_WIDE.pdf]

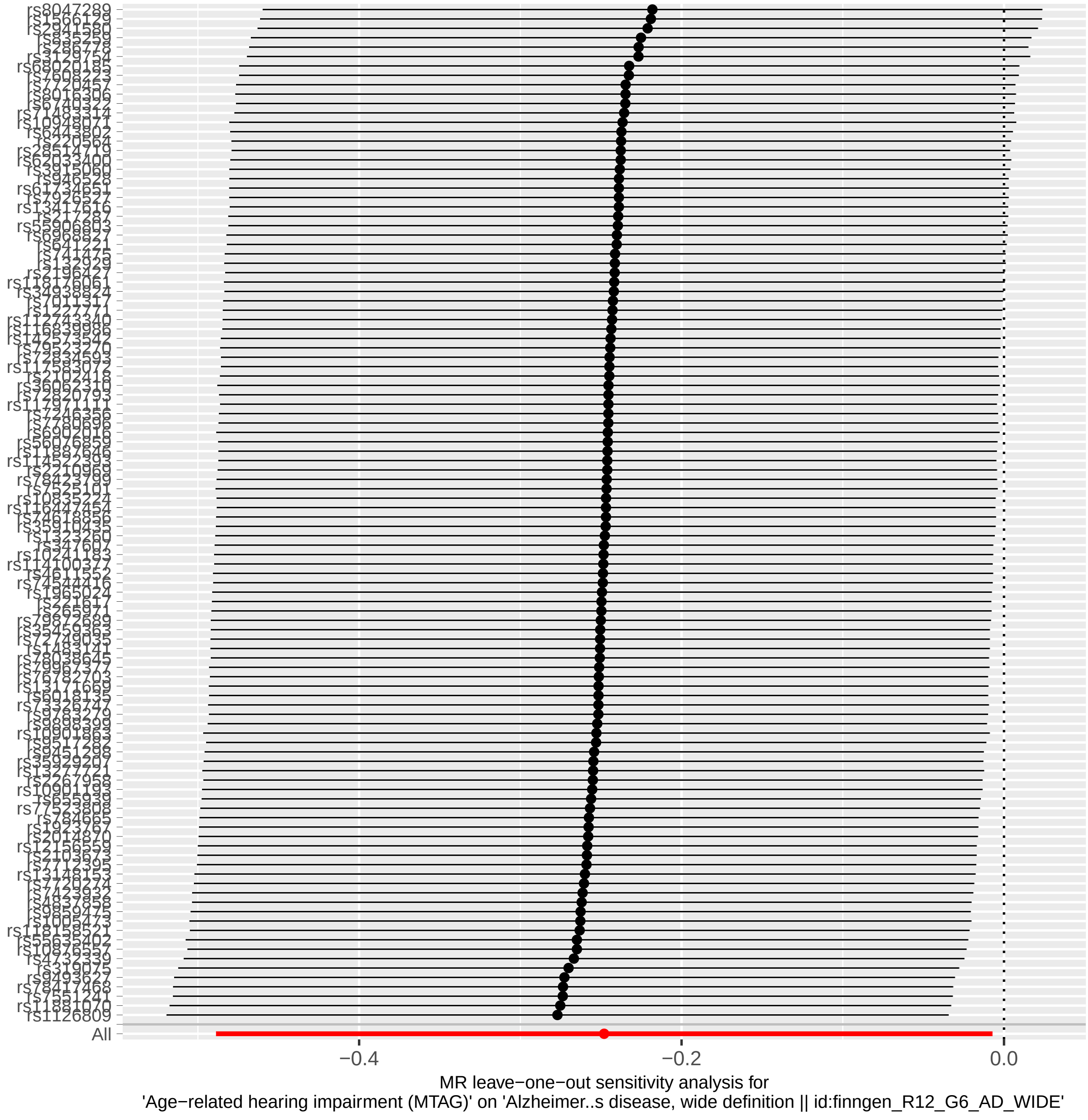

rs8047289
rs1126809
All
−0.4
−0.2
0.0
MR leave−one−out sensitivity analysis for
'Age−related hearing impairment (MTAG)' on 'Alzheimer..s disease, wide definition || id:finngen_R12_G6_AD_WIDE'
